# Quantifying multi‐institutional ADC measurement variability of 1.5 T MR‐Linacs: A phantom and in vivo study

**DOI:** 10.1002/mp.17739

**Published:** 2025-03-13

**Authors:** Madeline E. Carr, Kathryn E. Keenan, Michaela Beavan, Hilary Byrne, Satomi Higuchi, Amy Walker, Sarah Elliott, John Baines, Vikneswary Batumalai, Peter Metcalfe, Lois Holloway, Michael G. Jameson

**Affiliations:** ^1^ GenesisCare Sydney New South Wales Australia; ^2^ Centre for Medical Radiation Physics University of Wollongong Wollongong New South Wales Australia; ^3^ Liverpool and Macarthur Cancer Therapy Centres/Ingham Institute for Applied Medical Research Liverpool Australia; ^4^ National Institute of Standards and Technology Boulder Colorado USA; ^5^ School of Clinical Medicine, Medicine and Health University of New South Wales Sydney New South Wales Australia; ^6^ School of Mathematical and Physical Sciences University of Newcastle Newcastle New South Wales Australia; ^7^ Department of Radiation Oncology Olivia Newton‐John Cancer Wellness and Research Centre Austin Health Heidelberg Australia; ^8^ Department of Physical Sciences Peter MacCallum Cancer Centre Melbourne Australia; ^9^ Townsville Cancer Centre Townsville University Hospital Townsville Australia; ^10^ College of Science and Engineering James Cook University Townsville City Queensland Australia; ^11^ The George Institute for Global Health University of New South Wales Barangaroo New South Wales Australia

**Keywords:** accuracy, apparent diffusion coefficient (ADC), MR‐Linac, quantitative magnetic resonance imaging (qMRI), repeatability, reproducibility

## Abstract

**Background:**

Diffusion‐weighted imaging (DWI), a quantitative magnetic resonance imaging (qMRI) technique, has the potential to aid in disease characterization and treatment response monitoring. MR‐Linacs (MRLs) enable simultaneous DWI acquisitions during radiotherapy, uniquely aiding in the collection of large‐scale datasets for imaging biomarkers, such as the DWI‐derived apparent diffusion coefficient (ADC), without additional patient burden. However, the limited data reporting on variability in MRL scanner performance characteristics, and a lack of established clinical trial quality assurance (QA) procedures, are barriers to this route for biomarker validation.

**Purpose:**

This study aims to quantify the accuracy, intra‐scanner repeatability, and inter‐scanner reproducibility of ADC measurements across three MRLs in Australia in both a phantom and in vivo. These measurements will inform the feasibility of carrying out prospective multi‐center studies in Australia investigating ADC as a biomarker and form a core set of QA procedures and baselines to assess biomarker and sequence suitability.

**Methods:**

An isotropic diffusion phantom (at 0°C) and one healthy volunteer were scanned on three Unity MRLs (Elekta AB, Stockholm, Sweden). Standardized (QIBA Diffusion Profile) and anatomy‐specific DWI sequences, including sequences recommended by the MR‐Linac Consortium Imaging Biomarker Working Group, were used to image the phantom and volunteer. ADC maps generated using the MRL scanner software (inline ADC) and diffusion‐weighted (*b*‐value) images were exported from the scanner console. The latter was used to generate ADC maps using commercial software (offline ADC) for a separate comparative analysis. Performance metrics were computed for each sequence, including a coefficient of variation to assess between‐session intra‐scanner repeatability (CV_BS_) and inter‐scanner reproducibility (CV), for each phantom vial and contoured organ. Additionally, using the phantoms’ known ADC vial values, a percentage bias (bias) was calculated to determine ADC accuracy.

**Results:**

Phantom‐based measurements for the standardized QIBA sequence had intra‐ and inter‐scanner CV and bias well within recommended guideline (QIBA Diffusion Profile) tolerance limits of 2.2% and ±3.6%, respectively. All anatomy‐specific phantom DWI sequences were also within these tolerances, except for the cervix sequence at one site which showed an average intra‐scanner bias of +4.5%. Both accuracy and reproducibility for all sequences were worse for lower diffusivity vials measured in the phantom. Additionally, inline and offline ADC maps had high similarity with average percent differences of +0.2%. Volunteer‐based results had worse reproducibility, with the average inter‐scanner CV for the brain and pancreas sequences within 9.0%, however, reaching up to 27.1% for pelvis and abdomen sequences.

**Conclusions:**

This study demonstrated accuracy, intra‐scanner repeatability, and inter‐scanner reproducibility comparable to metrics reported in the literature, using both the phantom and volunteer datasets. The cervix sequence had the largest variability in both phantom and volunteer results and was recommended for further investigation. This study suggests that qMRI techniques utilizing DWI could be a viable option for future multi‐centered patient‐based studies utilizing Australian MRLs, with phantom‐based quality assurance recommended alongside patient imaging.

## 1 INTRODUCTION

Diffusion‐weighted imaging (DWI), a quantitative magnetic resonance imaging (qMRI) technique, has potential applications in disease characterization and treatment response monitoring.^1^, ^2^, ^3^, ^4^ The apparent diffusion coefficient (ADC) is a quantitative imaging biomarker (QIB) derived from DWI that measures water diffusivity in tissues, providing insights into tissue cellularity. Collecting large‐scale datasets of patient QIB values over each day of their treatment is typically a challenge within a conventional radiotherapy environment. Collecting this data requires patients to be moved from treatment machines to the diagnostic MRI, adding to patient burden and increasing clinical staff time and department resources to obtain these images.^5^, ^6^

Combining datasets from multiple centers is one way to increase the available data for examining changes in QIBs with treatment response. However, failure to conduct appropriate inter‐ and intra‐scanner qMRI quality assurance (QA) procedures to assess accuracy and variability limits the reliability of the patient‐derived QIB values in the collected datasets.^7^ Performing QA specific to quantitative imaging types such as DWI is a relatively new recommendation from the literature, and thus many past multi‐center in vivo DWI studies have not reported the implementation of such QA.^7^, ^8^ Performing this type of QA is now recommended before commencing any clinical trial using MRI QIBs.^9^

MR‐Linacs (MRLs) offer the unique capability to acquire DW‐images concurrently with each fraction of radiotherapy.^2^, ^6^, ^9^, ^10^, ^11^ This allows the efficient collection of extensive MRI QIB datasets without additional patient burden. These QIB datasets will help build the evidence required to enable clinical implementation of QIB‐based treatment decisions.^5^, ^9^, ^12^ However, there are particular hardware features of MRLs, such as split gradient coil design to facilitate the treatment beam, which could cause deviations in QIB measurements compared to diagnostic MRI scanners.^6^, ^10^, ^12^ Ensuring that MRL systems are reliable in generating QIBs is essential and should occur both before and during the collection of large datasets in clinical trials.^9^, ^11^

**FIGURE 1:**
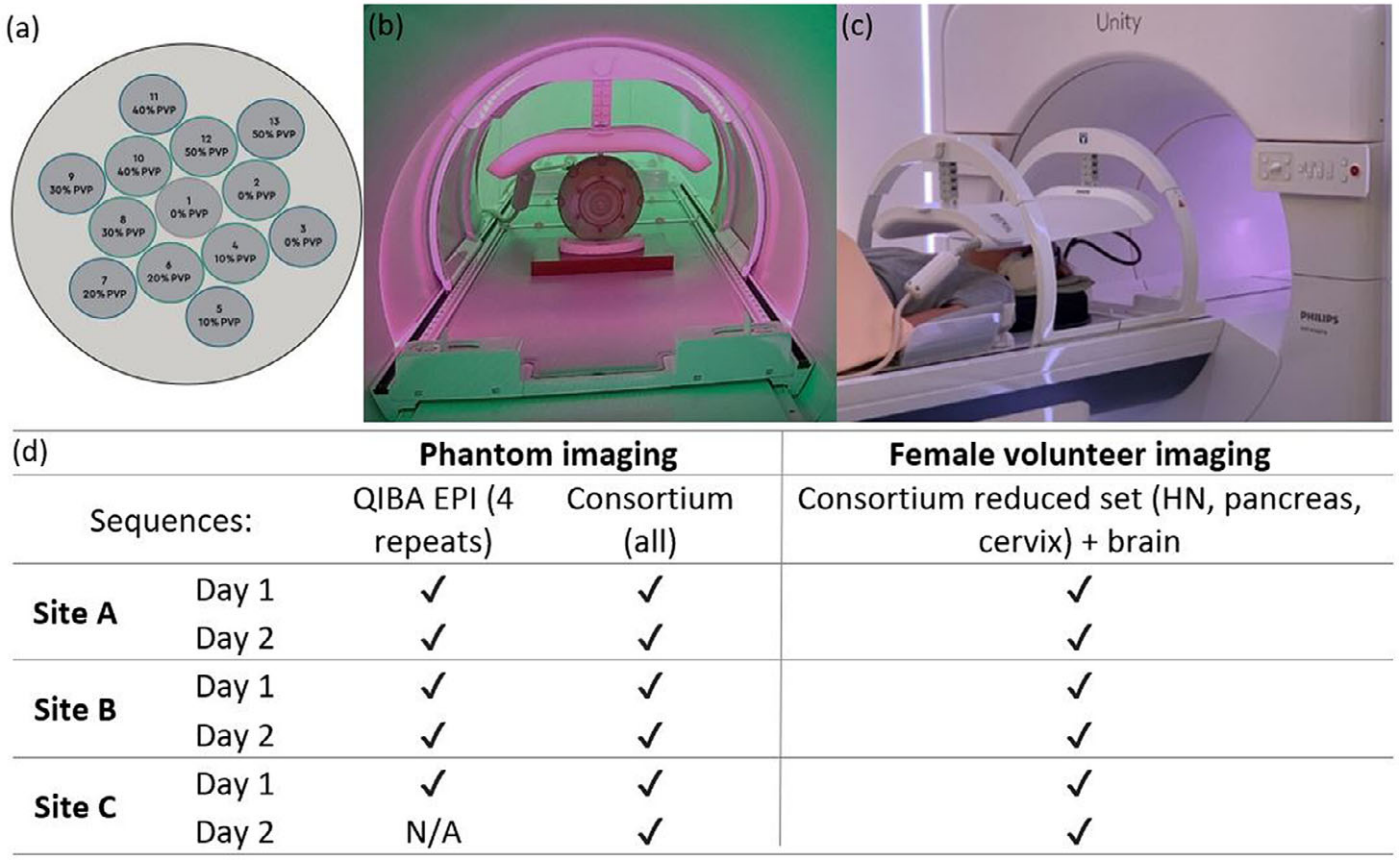
(a) Phantom schematic, highlighting vial number arrangement with polyvinylpyrrolidone (PVP) concentration, in its axial position. (b) Phantom axial positioning with body coil, solid water (plastic‐based material), and stabilizing foam for maintaining positional accuracy between the three sites. (c) Healthy volunteer setup for brain and head and neck (HN) Consortium sequence acquisition on one of the MRL systems. The body coil in use was positioned to encompass brain and HN sequences, but the size of the field of view changed in between sequences. (d) Phantom (*n* = 12) and volunteer (*n* = 6) datasets acquired and included (✔) for this study between the three MRL sites. Note: site C, day 2 dataset was acquired, however was not retrievable and thus not able to be included in the analysis.

There are several studies in the literature investigating the qMRI performance of Unity MR‐Linacs (Elekta AB, Stockholm, Sweden).^5^, ^10^, ^11^, ^12^, ^13^, ^14^ This includes investigating ADC variability using the Quantitative Imaging Biomarker Alliance (QIBA) recommended diffusion phantom (CaliberMRI, Boulder, CO, USA).^15^ As an overview, this phantom includes a central water vial surrounded by an inner and outer ring of vials of varying concentrations of polyvinylpyrrolidone (PVP) (Figure 1). Specifics on its design has been reported in the literature and has well‐established reference ADC values at 0°C.^7^, ^15^, ^16^ In an intra‐scanner setting, short‐term (ST) and between‐session (BS) repeatability of the central water vial ADC measured using coefficients of variation (CV) was found to be CV_ST_ < 3.3%,^5^, ^10^ CV_BS_ < 1.3%,^5^, ^10^, ^13^ and accuracy given by measurement percentage bias (bias) was found to be <+0.8%.^5^, ^13^

In one of only a few studies investigating in an inter‐scanner MRL setting, a phantom study by Kooreman et al. found ADC single time point reproducibility CVs between MRLs of 2.2%.^5^ Some in vivo studies have also been completed, with intra‐scanner repeatability and inter‐scanner (MR‐Simulator vs. MR‐Linac) reproducibility CV found to depend on organ type and/or sequence choice.^6^, ^10^, ^11^, ^17^ More recently, a study by Bisgaard et al. found organ delineation and ADC calculation methods to be factors leading to ADC variation, with the latter having a larger impact.^14^

This study aims to quantify the accuracy, intra‐scanner repeatability, and inter‐scanner reproducibility of ADC measurements of three Australian Unity MRLs. Unlike previous studies, this work novelly presents findings using all anatomy‐specific sequences recommended by the MR‐Linac Consortium^12^, ^18^ in addition to the standardized 1.5 T phantom sequences recommended by the 2019 QIBA Diffusion Profile.^7^, ^8^ This profile specifically outlines methods and performance tolerances (e.g., for bias and repeatability) required to ensure negligible contribution of technical errors to tissue measurements, allowing for prospective reproducible in vivo multi‐center trials involving ADC.^8^

Further, using a traveling volunteer, a subset of the sequences used for phantom imaging were performed in vivo to assess if performance is similar between phantom and volunteer measurements. Repeated phantom and in vivo measurements on the same day using the same sequences are rarely performed in the literature, and no such investigation has previously been conducted in Australia. This work adds to the current understanding of inherent MRL variation, demonstrates the potential for collaboration, and will support planning for future multi‐center trials across these Australian centers.

## 2 METHODS

### 2.1 Imaging

Imaging was performed using three 1.5 T MRI scanners (Philips, Amsterdam, the Netherlands), each embedded within the Unity MRL and housed in an Australian radiotherapy department. Each of the scanners, referred to as A, B, and C in this work, had the same software version (Marlin 5.3.31, Elekta AB, Stockholm, Sweden) installed at the time of imaging. The isotropic diffusion phantom at 0°C (phantom serial number: DP128‐A‐03‐0113), manufactured by CaliberMRI (CO, USA), was scanned on 2 consecutive days on each of the scanners. The phantom's temperature was established and maintained using an ice‐bath, as detailed in the literature.^8^, ^15^

The central water vial of the phantom was positioned at isocenter within the MRI bore. Image acquisitions were performed with the phantom in its axial position to match axial slice selections, as per the QIBA Diffusion Profile.^8^, ^19^ Stabilizing foam (∼1 cm thickness) with positioning markers for the external sagittal laser was placed on top of 3 cm of a plastic‐based material to raise the central vial to isocenter and maintain similar positioning between the three scanners (see Figure 1). Additionally, the standard Unity MRL body coil was adjusted to a consistently set height and positioned over the phantom to enhance image signal quality from the phantom.

On each day of measurement, the phantom was scanned four times sequentially using the QIBA echo‐planar imaging (EPI) diffusion sequence. This was a standardized single‐shot EPI (SS‐EPI) sequence, designed for 1.5 T Philips MRI of phantoms, with acquisition parameters listed in the QIBA Diffusion Profile.^8^ The phantom was also scanned using anatomy‐specific sequences obtained through the private MR Linac Unity user platform, provided by the MR‐Linac Consortium Imaging Biomarker Working Group.^12^, ^18^ These followed Consortium guidelines for ADC measurement as provided by Kooreman et al.^12^ and are hereafter referred to as the Consortium sequences. This included those designed for imaging brain, abdomen, and pelvis anatomies (i.e., head and neck [HN], lung, esophagus, pancreas, prostate, and cervix). A list of the key acquisition parameters can be found in Table 1.

**TABLE 1:** Acquisition parameters used for the QIBA Diffusion Profile, standard brain DWI protocol, and selected Consortium sequences which were used to image the phantom and the volunteer. All images were acquired axially and were single‐shot echo‐planar imaging (EPI) sequences.

Sequence/ Sequence parameter:	QIBA EPI	Brain ^v^	Head and neck (HN)	Lung	Esophagus	Pancreas/lymph	Prostate	Cervix/Rectum
**FOV (AP x RL x FH) (mm)**	220 × 220 × 125	281 × 230 × 105.6	300 × 300 × 120 or 300 × 300 × 235.3^v^	350 × 450 × 120	420 × 420 × 120	420 × 420 × 100	430 × 430 × 68	430 × 430 × 100
**Acq voxel size (AP x RL x FH) (mm)**	1.7 × 1.7 × 4	2.4 × 2.2 × 4.8	3.5 × 3.5 × 4	3.5 × 3.5 × 4	3.5 × 3.5 × 4	3.5 × 3.5 × 4	4 × 4 × 4	4 × 4 × 5
**Recon matrix**	256 × 256	352 × 352	192 × 192	288 × 288	240 × 420	240 × 240	224 × 224	224 × 224
**# Slices**	25	22	30	30	30	25	17	20
**Slice thickness (mm)**	4	4.8	4	4	4	4	4	5
**Slice gap (mm)**	1	0	0 or 3.8 ^v^	0	0	0	0	0
***b*‐values (s/mm^2^)**	0, 500, 900, 2000	0, 1000	0, 150, 500	0, 150, 500	0, 150, 500	0, 150, 500	0, 150, 500	0, 150, 500
**TR (ms)**	10 000	3806.5	4310.7 or 4308.3 ^v^	4542.7	4482.88	3759.8	3354.2	3520.9 or 4179.4 ^v^
**TE (ms)**	169.2	83.8	75.4	86.6	81.4	81.4	82.3	66.0
**BW (Hz/pix)**	1097	1360	2174	2233	2233	2233	2540	2532

*Note*: Volunteer scans only included the brain, HN, pancreas, and cervix sequences. ADC maps for Consortium sequences were generated using only two of the collected *b*‐values: 150 and 500 s/mm^2^, as per Consortium recommendations.^12^ The flip angle for all sequences was 90°, and as per the Consortium sequence acquisition parameters, the echo time (TE) and repetition time (TR) were set to “shortest.” As a result, the TE and TR could vary slightly between acquisitions at the three sites. Other acronyms include: BW = bandwidth, FOV = field of view. Lastly, ^v^ was used to indicate that the corresponding volunteer scan had differences in acquisition parameters compared to phantom acquisitions, or for the case of the brain, only volunteer acquisitions were performed.

Lastly, a 26‐year‐old healthy female volunteer was imaged on the same consecutive days as the phantom measurements at each site. This involved using a subset of the Consortium sequences (HN, pancreas, and cervix), plus one standard brain‐DWI sequence that was supplied on the MRL software. The volunteer took two 10 mg Buscopan tablets 30 min prior to each imaging session to reduce bowel and muscle motion during the scan. No bladder preparation was conducted. The volunteer provided informed consent, with imaging conducted as part of a project approved by the local ethics board at each site: HREC 015/20 for GenesisCare St Vincent's Hospital, HREC/88477/Austin‐2022 at Olivia Newton‐John Cancer & Wellness Centre Austin Hospital, and HREC/QTHS75997 for Townsville Hospital.

The volunteer was positioned by an experienced MR‐radiographer or MRL‐trained radiation therapist. The region of anatomy to be imaged, including head region for brain and HN sequences, abdomen for pancreas, and pelvis for cervix, was positioned at isocenter and the standard body coil was placed on top of the respective anatomy (Figure 1b). The field of view (FOV) was changed between sequences to ensure the correct anatomy was centered in the image. Acquisitions involved a survey scan for anatomic alignment, a 3D T1‐weighted image for subsequent contouring, and then the selected DWI sequences.

### 2.2 Image analysis

#### 2.2.1 Phantom

Both the DW‐images obtained at each *b*‐value, and the ADC maps generated from the MRL scanner software (inline ADC) from all acquired DWI sequences were exported from each Philips scanner console. All phantom‐based data analysis was centralized post‐acquisition and completed by a single physicist. The next step of analysis involved generating ADC maps (offline ADC), of both the QIBA EPI and Consortium diffusion sequences, using the sequences respective DW‐images in the commercial software qCal (CaliberMRI, CO, USA). qCal is the phantom manufacturer's recommended analysis software which is approved by QIBA for conformance testing.^20^ This software calculates the ADC using a least squares fit using the following formula for each pixel, where $S_{bx}$ and $S_{b0}$ are the values of the pixel in the DW‐image with *b*‐value of x and 0 s/mm^2^, respectively:

$$\;S_{bx}=S_{b0}\;*e^{-bx*\mathrm{ADC}}$$

Using qCal, a number of intra‐scanner QIBA profile conformance statistics were calculated using each sites’ day 1 image from the QIBA EPI diffusion sequence. This included calculating the intra‐scanner short‐term (ST) repeatability (CV_ST_) and repeatability coefficient (RC_ST_), and also signal to noise ratio (SNR), all using four repetitions of the sequence performed on the same day.

The calculations performed using the first repetition of the QIBA EPI sequence included determining the bias by comparing the measured ADC to each vial's reference ADC value for an accuracy measurement. Particularly, the ADC of the central water vial of the phantom was the focus for this study (vial 1 in Figure 1a), with a known characterized ADC value of 1.109 µm^2^/ms at 0°C.^15^ However, all vials were analyzed using the software, including for bias calculations and additionally for determining the linearity between measured and reference ADC vial values with a coefficient of determination (*R* ^2^). Precision (CV_p_) and *b*‐value dependency (Dep_b_) were also calculated in the software using the first repetition only, along with intra‐scanner between‐session (BS) repeatability (CV_BS_ and RC_BS_). This is where the latter two metrics were assessed by using the standard deviation (SD) and mean of repeat first measurements on 2 consecutive days.

An inbuilt qCal software function (i.e., “Multi‐Study Report”) was used to calculate the inter‐scanner reproducibility (CV) and average bias in each of the phantom vials. This involved first using the day 1 QIBA EPI sequence intra‐scanner average %bias values for each vial, as calculated from the 4× daily repetitions for each scanner, and then calculating the average inter‐scanner CV and mean bias between the three MRLs. The full list of QIBA Profile calculations used in the intra‐ and inter‐scanner statistical analysis of this study is detailed in Table S1, consistent with methods described in the literature.^5^, ^7^, ^8^, ^19^, ^21^ Performance metrics were compared to QIBA Diffusion Profile tolerance limits (tolerances outlined in Table 2).^8^

**TABLE 2:** QIBA Diffusion Profile tolerances and corresponding results for QIBA EPI diffusion sequence for each site, including inter‐scanner accuracy, short‐term coefficient of variation for repeatability (CV_ST_), between‐session repeatability (CV_BS_), linearity, random error, SNR, and *b*‐value dependence (tests A → G).^8^

Test	Performance metric	Tolerance	Site A	Site B	Site C
**A**	|bias (%)|	≤3.600	−0.026	−0.194	+0.047
**B**	$RC_{\mathrm{ST}}$ (µm^2^/ms) $CV_{\mathrm{ST}}\;\left(\%\right)$	≤0.015 ≤0.5	0.012 0.4	0.009 0.3	0.006 0.2
**C**	$RC_{\mathrm{BS}}$ (µm^2^/ms) $CV_{\mathrm{BS}}\;\left(\%\right)$	≤0.065 ≤2.2	0.001 0.0	0.007 0.2	N/A N/A
**D**	$R^{2}$ $\mathrm{slope}\;(\beta_{1})$	> 0.9 0.95 ≤$\;\beta_{1}$≤ 1.05	1.0 1.00	1.0 1.00	1.0 0.99
**E**	$CV_{P}$ (%)	<2	4.4	5.2	4.9
**F**	SNR *b =* 0 s/mm^2^	≥50 ± 5	79	75	71
**G**	$\mathrm{Max}\;\mathrm{De}p_{b}$(%) Max *b*‐value pair (s/mm^2^)	<2 0, 500, 900 or 2000	1.3 500, 2000	0.8 500, 2000	1.2 500, 2000

*Note*: All metrics were calculated using the offline automated analysis software, qCal, using ADC measurements for the central water vial of the phantom.

For the Consortium sequences, three DW‐images using three individual *b*‐values were acquired during acquisition as shown in Table 1. However, only the 150 and 500 s/mm^2^ *b*‐value DW‐images were used to generate both the inline and offline ADC maps. This was completed to align with the volunteer datasets, which exclude the 0 s/mm^2^ *b*‐value DW images to mitigate the pseudodiffusion effect, following recommendations in the literature.^6^, ^12^, ^22^ Note that removal of the 0 s/mm^2^ *b*‐value images from the offline ADC maps was only possible through close collaboration with the qCal vendors.

The phantom‐based analysis for these sequences used the inline ADC maps and an in‐house developed Python script. This script calculated each sequence's average intra‐scanner bias and intra‐scanner repeatability (CV_BS_), and also inter‐scanner average bias and reproducibility (CV), as per the QIBA Diffusion Profile statistical analysis methods.

#### 2.2.2 Inline versus offline ADC maps

The day 1, repetition 1, offline generated ADC maps of both the QIBA Diffusion Profile and each Consortium sequence from one MRL site were compared with the corresponding inline maps using an in‐house developed Python script. This involved placing manual regions of interest (ROIs) over vial 1 of ∼1 cm diameter on the central three slices of the ADC maps and comparing the ADC value within the ROI across the three slices. Offline maps were generated with units of µm^2^/s and thus needed scaling for comparability with inline maps with units µm^2^/ms.

From the literature, it is recommended that images are unscaled prior to ADC analysis (especially for Philips scanners) to ensure accurate comparisons between sequences and scanners.^8^, ^14^, ^23^ In this study, the rescaling intercept and slope were accounted for in the offline ADC map generation, and also in the analysis of inline ADC maps.

#### 2.2.3 Volunteer

For the volunteer images, the volumetric T1‐weighted images and inline ADC maps were exported from the scanner consoles, consistent with department protocols for patient imaging. These were uploaded to MIM (MIM Software Inc., Cleveland, OH, USA) and a single radiation therapist contoured several organs of interest in all the T1‐weighted datasets. All remaining analysis for the volunteer dataset was again centralized and completed by the same physicist as per the phantom data.

These contours were rigidly aligned to the inline ADC maps, and only organs fully contained in the ADC FOV were included in the analysis. This involved extracting the average ADC value from within each organ using MIM. ADC statistics were calculated, including the average organ ADC value and intra‐scanner repeatability (CV_BS_) using the 2 consecutive days measurements per MRL, and inter‐scanner reproducibility (CV) comparing day 1 organ ADC measurements between scanners.

## 3 RESULTS

A total of 12 phantom datasets were collected including the QIBA EPI sequence and the Consortium sequences, each repeated over 2 consecutive days at the three MRL sites (see Figure 1). At site C, however, the second day of QIBA dataset measurements was not retrievable, resulting in 11 datasets for analysis. Additionally, six datasets of the volunteer were acquired across the three MRL sites. This included the standard brain DWI protocol and the Consortium sequences, each collected on the same 2 days as the phantom imaging.

For the volunteer images, only some organs were fully contained inside the ADC map FOV for certain day acquisitions and thus enabled ADC value extraction from the contours (see Table S2). Completely contained organs included the brain; brainstem; cerebellum and orbits; kidneys; and cervix, femurs, rectum, and uterus.

### 3.1 Phantom imaging

#### 3.1.1 Phantom: QIBA diffusion profile measurements

A summary of results obtained using the QIBA EPI sequence compared to the Diffusion Profile tolerances is provided in Table 2. Specifically, all results, except for the precision (CV_p_), were within the QIBA Diffusion Profile limits. Typical ADC maps of the phantom, generated from acquiring the QIBA EPI sequence, can be observed in Figure S1.

Table 3 presents the ADC bias and CV as a function of PVP concentration (%). Although only vial 1 was required for QIBA Diffusion Profile tests,^8^ these data were still considered valuable given the wide range of ADC values found in the human body. Generally, as the PVP concentration in the vials increased (i.e., lower diffusivity), accuracy and reproducibility decreased, with the worst bias and CV reaching +9.1% and 5.9%, respectively, between the most concentrated vials (12 and 13).

**TABLE 3:** NIST reference ADC values^15^ and average inter‐scanner ADC bias and CV between the three MRL sites using day 1 imaging sessions.

Vial number	NIST reference ADC (µm^2^/ms)	Av. inter‐scanner ADC bias (%)	Inter‐scanner CV (%)
**1^c^**	1.109	−0.06	0.10
**2^i^**	1.109	0.67	0.43
**3**	1.109	1.71	0.53
**4^i^**	0.817	1.58	0.20
**5**	0.817	2.59	0.20
**6^i^**	0.579	1.48	0.11
**7**	0.579	2.35	0.39
**8^i^**	0.380	4.46	0.34
**9**	0.380	6.26	0.18
**10^i^**	0.220	5.25	0.42
**11**	0.220	5.25	2.54
**12^i^**	0.110	7.54	5.86
**13**	0.110	9.11	2.68
**Av All**	–	3.71	1.07

*Note*: In each case, the average over each of the phantom vials is given, along with identifying if the vial is a central (^c^) water vial, or one of the six surrounding inner‐ring (^i^) vials (see Figure 1, vials: 2, 4, 6, 8, 10, and 12). Reference ADC values can vary depending on the serial number of the diffusion phantom.

#### 3.1.2 Phantom: Inline versus offline ADC maps

**FIGURE 2:**
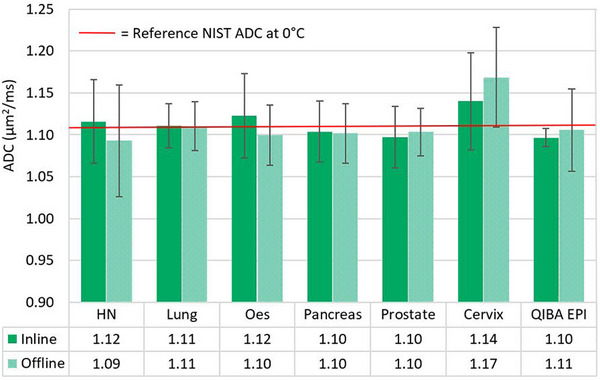
Inline versus offline ADC results for site A, day 1, vial 1, for all consortium sequences plus the first repetition of the QIBA profile sequence. Error bars represent the standard deviation in the mean ADC measurements between regions of interest in the central phantom slices. Also, the reference NIST ADC of this vial (1.109 µm^2^/ms) is provided in red for comparison to the known standard at 0°C.

For both QIBA Profile and Consortium sequences, the inline and offline ADC maps had high similarity in ADC values (see Figure 2). A visual comparison between the inline and offline QIBA EPI‐generated ADC maps can be seen in Figure S1. Quantitatively, for site A, day 1 (and repetition 1 for the QIBA EPI sequence), the percentage difference in vial 1 ADC values between inline and offline ADC maps ranged between −2.4% to +2.1% across QIBA EPI and all Consortium sequences (average between all sequences = +0.2%). Given the consistency of results, inline maps were used for the remainder of the Consortium results presented. Additionally, the inline and offline Consortium cervix sequence ADC maps showed the largest deviation from the known NIST reference value, with percentage differences of up to 2.8% and 5.4%, respectively.

#### 3.1.3 Phantom: Consortium sequences

Table 4 summarizes the average intra‐ and inter‐scanner ADC values found for vial 1 across each Consortium sequence. In most cases, site C exhibited the most repeatable ADC, as indicated by the lowest CV_BS_, compared to the corresponding inter‐scanner CV. In contrast, sites A and B showed variability in CV_BS_ compared to the inter‐scanner CV, depending on the sequence type. This suggests higher day‐to‐day variability on these scanners, particularly at site B, as found in the QIBA Diffusion Profile results (Section 3.1.1).

**TABLE 4:** Phantom imaging intra‐ and inter‐scanner average ADC values for the central water vial and corresponding repeatability, reproducibility (CV_BS_ and CV), and bias recorded for each Consortium sequence.

Sequence	Mean intra‐scanner ADC (µm^2^/ms) ± SD CV_BS_ [bias (%)]			Mean inter‐scanner ADC (µm^2^/ms) ± SD CV [bias (%)]
	Site A	Site B	Site C	
**HN**	1.11 ± 0.01 0.57% [+0.04]	1.11 ± 0.00 0.18% [0.00]	1.12 ± 0.00 0.03% [+0.96]	1.12 ± 0.00 0.33% [+0.59]
**Lung**	1.11 ± 0.00 0.13% [+0.03]	1.10 ± 0.00 0.03% [‐0.40]	1.12 ± 0.00 0.20% [+0.58]	1.11 ± 0.00 0.34% [+0.03]
**Esophagus**	1.12 ± 0.01 0.53% [+0.70]	1.10 ± 0.01 0.57% [−0.63]	1.11 ± 0.00 0.44% [‐0.19]	1.11 ± 0.01 1.04% [−0.20]
**Pancreas**	1.11 ± 0.00 0.13% [−0.34]	1.11 ± 0.01 0.94% [−0.19]	1.11 ± 0.00 0.02% [−0.21]	1.10 ± 0.00 0.40% [−0.60]
**Prostate**	1.11 ± 0.01 0.93% [−0.16]	1.11 ± 0.00 0.33% [−0.17]	1.10 ± 0.00 0.18% [−0.47]	1.10 ± 0.00 0.25% [−0.75]
**Cervix**	1.16 ± 0.02 1.63% [+4.51]	1.10 ± 0.02 1.42% [−0.51]	1.14 ± 0.00 0.42% [+3.15]	1.13 ± 0.01 0.86% [+2.14]

*Note*: For the intra‐scanner averages and standard deviations (SDs), these performance metrics were obtained over 2 consecutive days of phantom measurements. For the inter‐scanner metrics, these were obtained between day 1 phantom measurements at each MRL site.

The maximum intra‐scanner CV_BS_ and bias were 1.6% and +4.5%, respectively, both observed for the cervix sequence at site A. This bias metric was outside the Diffusion Profile limits of ±3.6%. Across all sites, the cervix sequence showed the largest inaccuracies and poorest repeatability.

**FIGURE 3:**
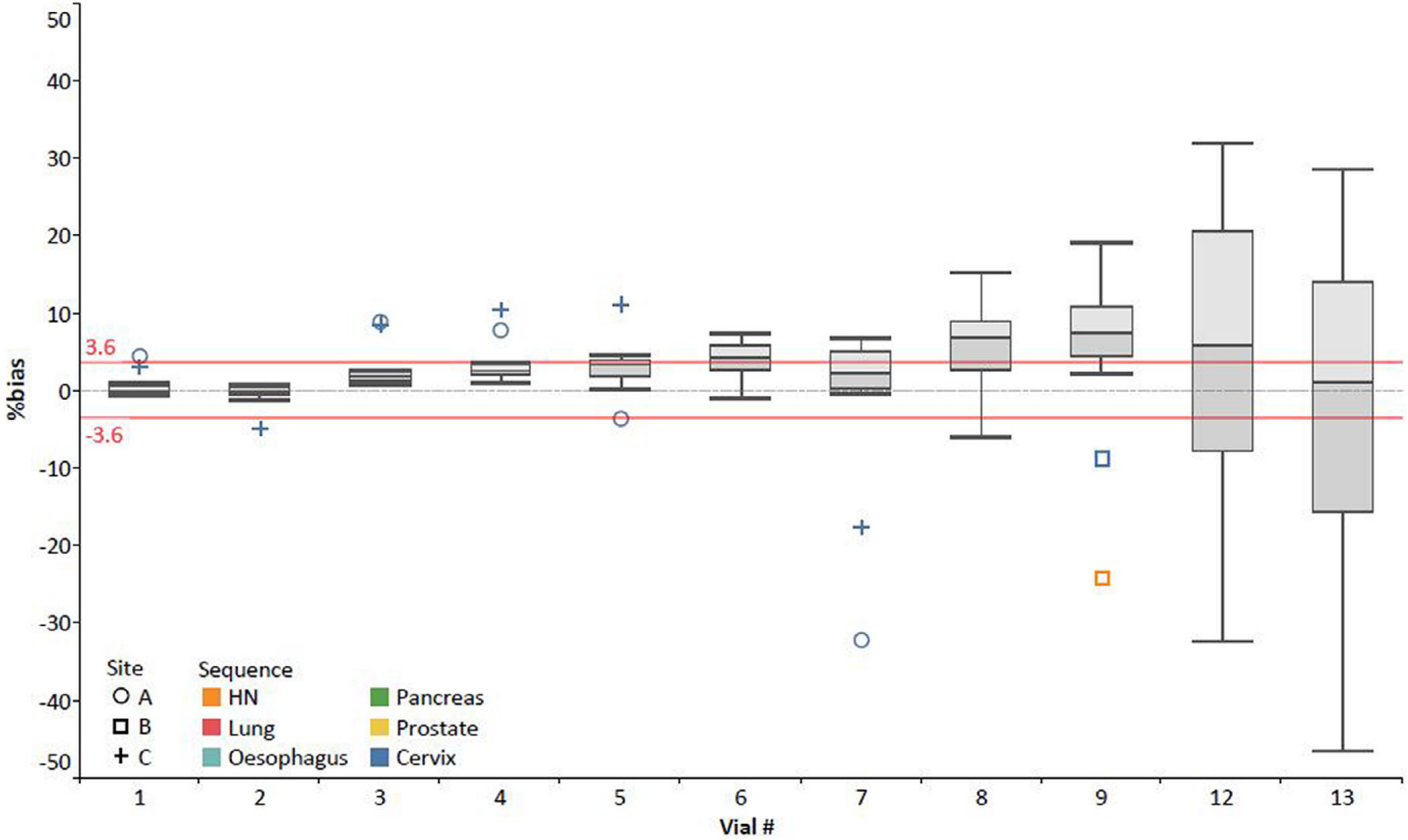
Intra‐scanner %bias values recorded between the three MRL sites for each vial within the phantom, for all six Consortium sequences tested, presented as a box and whisker plot with relevant median and interquartile ranges shown. The reference QIBA Diffusion Profile %bias tolerance of ±3.6% has been added. However, this tolerance is only required for the central water vial (vial 1) for QIBA Profile conformance testing. Note that vials 10 and 11 %bias results were removed as all sequences (excluding cervix and prostate) had significant signal deficits in these vials on the ADC maps and thus were assumed not to be real measures of bias (i.e., up to +303%) (see Figure S2).

Observing the inter‐scanner vial 1 results in Table 4, all sequences performed well, with high inter‐scanner reproducibility (CV ≤ 1.0%) and accuracy (bias ≤±0.75%, excluding the cervix sequence). The cervix sequence demonstrated less accuracy across the scanners, with an average bias of +2.14%, though still within Diffusion Profile tolerances (<±3.6%). This is also highlighted in Figure 3, where the ADC recorded for the cervix sequence diverged from the other sequences, particularly for non‐water‐equivalent vials.

Figure 3 also shows that, in general for the Consortium sequences, both accuracy (|bias| values) and reproducibility (indicated by the spread in bias values) decreased with higher PVP concentrations. By visual inspection, the phantom images acquired were noisier and subject to larger distortions around the higher PVP concentration vials (Figure S2). When calculating the CV across the full vial and Consortium sequence range, the CV was <2% for all water‐equivalent vials (vials 1–3) and <10% for vials 1–8 (or <5% excluding the cervix sequence).

### 3.2 Volunteer imaging

#### 3.2.1 Volunteer: Intra‐ and inter‐scanner

Qualitatively, the image quality of the volunteer scans for the Consortium sequences was noisy, and in some cases, it was difficult to distinguish anatomical features. Sample images have been included in Figure S3. Overall, the intra‐scanner volunteer‐based ADC results demonstrated good performance metrics (Table 5).

**TABLE 5:** Volunteer imaging intra‐ and inter‐scanner mean ADC and standard deviation (SD) values found for each contoured organ for the standard brain DWI protocol and Consortium pancreas and cervix sequences.

Sequence	Organ	Mean intra‐scanner ADC ± SD (µm^2^/ms) CV_BS_			Mean inter‐scanner ADC ± SD (µm^2^/ms) CV
		Site A	Site B	Site C	
**Brain**	**Brain (whole)**	0.77 ± 0.02 2.6%	0.81 ± 0.30 N/A	0.89 ± 0.37 N/A	0.83 ± 0.04 5.21%
**Brain**	**Brainstem**	0.79 ± 0.01 0.64%	0.86 ± 0.26 N/A	0.78 ± 0.21 N/A	0.81 ± 0.04 4.68%
**Brain**	**Cerebellum**	0.68 ± 0.00% 0.00%	0.74 ± 0.23 N/A	0.75 ± 0.20 N/A	0.72 ± 0.01 4.27%
**Brain**	**Orbits**	2.1 ± 0.03 1.19%	2.22 ± 0.27 N/A	1.95 ± 0.73 N/A	2.10 ± 0.11 5.32%
**Pancreas**	**Kidneys**	1.59 ± 0.12 7.55%	1.77 ± 0.02 1.13%	1.79 ± 0.51 N/A	1.68 ± 0.15 8.96%
**Cervix/ Rectum**	**Cervix**	1.73 ± 0.10 5.51%	1.64 ± 0.45 27.3%	1.89 ± 0.11 6.06%	1.67 ± 0.35 20.94%
**Cervix/ Rectum**	**Femurs**	0.61 ± 0.03 4.49%	0.67 ± 0.04 5.53%	0.62 ± 0.02 3.09%	0.63 ± 0.56 8.82%
**Cervix/ Rectum**	**Rectum**	1.07 ± 0.08 7.48%	1.45 ± 0.51 35.27%	1.7 ± 0.06 3.39%	1.28 ± 0.35 27.1%
**Cervix/ Rectum**	**Uterus**	1.53 ± 0.03 1.96%	1.86 ± 0.12 6.27%	1.79 ± 0.07 3.83%	1.65 ± 0.11 6.58%

*Note*: Intra‐scanner results were averaged over the 2 consecutive measurement days, where available. Given sites B and C had some organ contours outside the ADC map field of view on day 2 (Table S2), these organs have mean ADC values reported as day 1 measurements only and thus no corresponding CV_BS_. Additionally, SDs for these organs were calculated as the variation in ADC values per pixel within the contour. Inter‐scanner mean ADC and SD were calculated using day 1 measurements per organ from all three sites.

Specifically, the overall intra‐scanner CV_BS_ was found to be ≤2.6% for organs imaged using the standard brain DWI protocol at site A, and <7.6% for kidneys between sites A and B which used Consortium pancreas sequences. However, for organs situated in the pelvis region (which are more prone to volume/motion changes) and imaged using the cervix sequence, larger CV_BS_ values were observed, with the rectum showing a CV_BS_ of up to 35.3%.

In general, these pelvis organs also exhibited greater volume changes between contours in the corresponding datasets. Given the large variability in volumes between organs (e.g., orbits vs. whole brain), the magnitude of the percentage differences in volume changes between intra‐measurements was compared to the measured CV (data not shown). However, no linear correlation was found (*R* ^2^ < 0.015).

**FIGURE 4:**
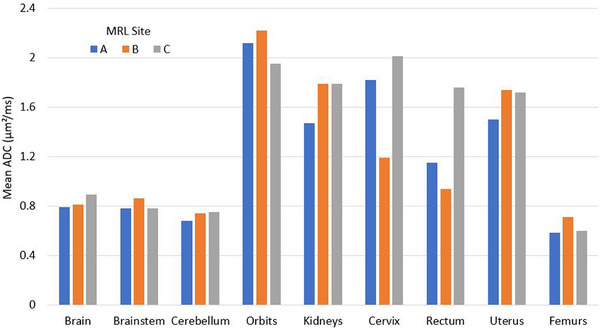
Variation of the ADC values measured across all three MRL sites (day 1 only) for the organs of interest.

When examining the inter‐scanner CV results based on day 1 measurements in Table 5, the CV increased substantially for all brain and abdominal organs compared to their intra‐scanner counterparts, as expected. Specifically, the CV was ≤5.32% for the brain and ≤8.96% for the pancreas sequence‐imaged organs, respectively. However, similar to the intra‐scanner results, there was significant variability in the inter‐scanner ADC values for organs imaged using the cervix sequence (as seen in Figure 4). The maximum CV recorded was 27.1% for the rectum, which was less than the intra‐scanner CV_BS_ maximum of 35.27% for the rectum.

## 4 DISCUSSION

MRLs have the capacity to generate large qMRI datasets, crucial for providing the evidence needed to integrate qMRI into radiotherapy for purposes such as monitoring treatment response and adapting treatment plans. However, recent studies have highlighted the need for phantom and in vivo validation testing before gathering large‐scale qMRI datasets.^9^, ^24^ The main purpose of this study was to investigate the ADC accuracy, intra‐scanner repeatability, and inter‐scanner reproducibility of 1.5 T Unity MRLs using a traveling phantom and volunteer. This is the first multi‐center MRL study conducted in Australia, and also the first demonstration of in vivo imaging using all the recommended sequences from the MR‐Linac Consortium working group, performed on both a phantom and traveling volunteer.

As noted, the QIBA Diffusion Profile has a set list of performance metrics that are recommended to be met for scanners involved in multi‐center trials to ensure ADC accuracy and reproducibility are maintained.^8^ In this study, all three MRLs demonstrated that nearly all intra‐scanner performance metrics were well within the Diffusion Profile tolerance limits. These results are consistent with the existing literature.^5^, ^10^, ^13^ High inter‐scanner reproducibility was also found using QIBA sequences. The average CV was 0.1% in the central water vial, and the average CV (and range) was 1.1% (0.1%–5.9%) when measured over all vials (Table 2). This was similar to inter‐scanner CV magnitudes reported in the literature for the QIBA sequences: median CV (and range) over all vials being 2.2% (0.6%–12%).^5^

One QIBA Diffusion Profile metric was out of tolerance (<2%): the intra‐scanner precision (CV_P_ = 4.4%–5.2%), similar to previous 1.5 T MRL studies reporting CV_P_ values of 5%–9%^5^ and 2.6%–3.6%.^10^ The literature suggests that using an 8‐channel body coil instead of the QIBA Diffusion Profile recommended head coil on Unity systems could affect sensitivity and thus this metric.^5^, ^6^ Kooreman et al. found that sequence parameter adjustments, such as lowering the *b*‐value, reducing echo time, and increasing voxel size, improved SNR and thus CV_P_.^5^

One limitation of this study is that it did not adhere to the most recent QIBA Diffusion Profile guidelines, as these were published after the data acquisition for this manuscript was completed. A key difference, between the acquisition protocols in the 2019 Profile^8^ and the latest 2022 Profile,^25^ is the use of five *b*‐values: 0, 500, 1000, 1500, and 2000 s/mm^2^. It is recommended that future studies should incorporate the updated Profile recommendations, including this change in phantom imaging acquisition parameters.

In multi‐center investigations and clinical trials, there are many sources of potential ADC variability that can limit the use of ADC as a reliable biomarker in a radiotherapy setting. Variability in ADC measurements between sites can stem from differences in setup or acquisition parameters,^4^, ^13^, ^14^ or from differences in image post‐processing analysis methods.^14^, ^26^ This study minimized setup and acquisition variability by using the same standardized sequence list, MRL type, phantom, and setup aids.

Post‐processing variability was minimized by using inline maps generated with the same calculation algorithm and software version across all three MRLs. Inline maps were visually comparable to offline maps, with the largest ADC difference observed in the central water vial of 0.03 µm^2^/ms. This difference agrees with the 0.02 µm^2^/ms variation reported by Carr et al. using the QIBA Diffusion Profile sequences on a Siemens 3 T radiotherapy dedicated MRI scanner.^19^

To the best of the authors’ knowledge, this study is the first to publish on the use of qCal software, which enabled automated ROI placement and analysis for the QIBA Diffusion Profile sequences, reducing potential user‐related variability in the QIBA Profile analysis. The use of centralized data processing through offline analysis aligns with literature recommendations for multi‐center diffusion studies.^14^, ^26^ However, comparing inline and offline ADC maps validated both methods for generating accurate ADC maps for both the Consortium and QIBA diffusion sequences, supporting the use in this case of the inline maps for in vivo based analysis, which was the preferred method for these MRL departments.

Comparing the phantom images taken with Consortium and QIBA sequences, it can be observed that the Consortium images are noisier, with a much larger FOV, and subject to larger geometrical distortions (Figures S1 and S2). However, excluding cervix, all other sequences had intra‐scanner performance metrics within Diffusion Profile tolerances (Table 4), suggesting sufficient accuracy, repeatability and reproducibility of the Consortium sequences for each scanner. The cause of the cervix sequence performance divergence would need to be further investigated prior to its recommendation for use in a clinical trial. The main differences in acquisition parameters from Table 1 include a larger slice thickness of 5 mm and the lowest TE of 66 ms. These parameters could have impacted diffusion sensitivity, leading to higher measured noise, and thus contributed to increased variability in datasets measured using the cervix sequence.

This study found that bias and reproducibility degraded with lower ADC reference values, consistent with the literature.^5^, ^13^, ^19^ This could be attributed to factors like insufficient SNR, where even small noise fluctuations can significantly impact the ADC curve fitting process,^5^, ^10^, ^12^ or increased probability of susceptibility‐induced distortions near the highly concentrated PVP vials due to their relative anterior position during axial imaging (Figure S1).^19^ It should be noted that using SS‐EPI sequences in general has several disadvantages, such as being prone to such susceptibility artefacts and also eddy currents, which can induce geometric distortions.^7^, ^11^ This could have affected the derived ADC from the phantom datasets, as well as the volunteer image datasets, especially for regions in the phantom and body positioned further than 7 cm from isocenter.^12^

There were large variations in Consortium sequence bias metrics in phantom vials 10 and 11, which were found to be sequence (and thus likely acquisition parameter) dependent rather than MRL site dependent. Observing Figure S2, it can be assumed that the deviations in these vials were not a measure of true ADC, but rather incorporate pixels with inadequate SNR. Prostate and cervix sequence images do not exhibit this effect, potentially due to higher SNR caused by the larger acquisition voxel sizes, smaller reconstruction matrix, and/or lower TRs. However, it should be noted that only vial 1 is required for QIBA Profile conformance testing. Also, vials 11–13 have reference ADC values outside the physiological range and are thus would not be considered of significant concern for prospective multi‐center trials.^19^, ^27^

For the volunteer imaging, both intra‐scanner repeatability and inter‐scanner reproducibility in vivo measures were acquired, as per recommendations in the literature.^7^, ^8^, ^9^ The specific ADC values of organs were less critical in this study. Instead, the focus was on the intra‐ and inter‐scanner variability. As expected, inter‐scanner reproducibility CV values (Table 5) were higher than intra‐scanner repeatability CV_BS_ for most sequences and organs, matching existing literature.^10^

In particular, intra‐/inter‐scanner volunteer imaging results showed a maximum CV_BS_/CV of 2.6%/5.3% for the brain and 7.6%/9.0% for the pancreas sequences. Most Unity MRL intra‐scanner ADC repeatability (either same day or between days) investigations have been focused on brain/HN anatomy, with comparable magnitude repeatability findings using SS‐EPI sequences with CV_BS_ up to 1.8% for healthy brain tissues^6^ and 6.72% for parotoid glands.^10^ Further, pelvis organs imaged using the cervix sequence had larger variations than previously reported, with a maximum CV_BS_/CV of 35.3%/27.1%.

The larger deviations in ADC observed in pelvic and abdominal organs could be attributed to the greater motion and anatomical changes in these regions compared to the brain. However, repeatability results showed changes in pelvic organ ADC values (Table 5), differing from the study by Ingle et al. who showed stable ADC values for healthy tissue (ovaries and seminal vesicles) between imaging sessions.^17^

The overall image quality of the volunteer ADC maps was poor (Figure S3). While T1 anatomical images were sufficient for contouring, identifying anatomical features on the ADC maps was difficult, even when the contours were overlaid. Variability in volunteer setup, shimming area, and FOV selection across sites, due to different staff and training, led to some organs being partially or fully excluded from some of the ADC maps.

Contour variability is a well‐known issue in radiotherapy and the past literature has shown it can impact ADC studies.^14^ The block‐based appearance of the cervix contour in the cervix Consortium sequence image observed in Figure S3 suggests the creation of this contour could have impacted ADC variability. Additionally, the contours were generated on the T1‐weighted dataset while ADC measurements were derived from the ADC maps. Thus, another limitation was possible misregistration between ADC maps and T1‐weighted images. Although ADC maps were acquired in the same session as the T1 images, susceptibility‐induced distortions or small movements, especially in the rectum, could cause geometric variation and ADC variability, and thus impact intra‐ and inter‐scan CV (see Figure S3).

However, the overall effect of contour variability was minimized by using a single user to contour all volunteer datasets. Although intra‐ and inter‐scanner differences in organ volumes were briefly examined, no clear link was found between volume changes and ADC variations. For future studies, providing more detailed imaging protocols (e.g., FOV selection) beyond acquisition parameters and training to staff are recommended for a more reliable inter‐scanner reproducibility measure. With such standardization, it is hypothesized that it is possible to normalize volunteer or patient‐based ADC values by accounting for differences in bias measured with a phantom during inter‐site QA comparisons.

Phantom‐based studies generally underestimate the ADC variability that could occur in patients’ due to their higher SNR, absence of patient motion, and lack of tissue complexities.^7^, ^9^, ^13^ While phantom results in this study adhered to QIBA conformance tolerances, suggesting adequate accuracy and reliability, volunteer results showed larger ADC variability, especially in regions outside of the brain.

Thus, relying only on phantom measurements could lead to an inflated sense of accuracy or reproducibility. Alternatively, performing only in vivo imaging could lead to the assumption that physiological changes occurred between imaging sessions rather than ADC changes inherently caused by the scanner or differences existing between scanners. This highlights the importance of implementing both phantom and in vivo‐based QA to ensure data reliability in prospective single or multi‐center clinical trials involving patient QIBs like ADC.

Based on the findings of this study, the authors recommend implementing a robust ADC QA program for MRL Unity systems, especially those involved in single‐ or multi‐site studies with quantitative ADC measurements. Guidance can be drawn from the MR‐Linac Consortium Biomarker Working Group,^9^ which aligns with this work's approach of acquiring phantom and in vivo datasets to assess bias and repeatability, respectively, before commencing clinical trials. Although optimal QA frequency first requires longitudinal studies for individual scanners, the literature suggests annual ADC QA testing, supplemented by additional testing after major MRI hardware or software upgrades.^19^ These assessments should include QIBA Profile testing and evaluation of any sequences intended for use in prospective trials.

This study has demonstrated inter‐scanner QA of ADC as a QIB; suitable for informing multi‐center data collection. Future studies could explore improved sequences to reduce variation, validate findings by expanding healthy volunteer and patient cohort testing, and explore the potential of normalization methods between phantom and in vivo results. This work provides benchmarks to assist in QIB validation of new machines as installed, allowing them to contribute data to prospective clinical trials in the region and globally.

## 5 CONCLUSIONS

Overall, the phantom‐based intra‐ and inter‐scanner ADC measurements performed well in comparison to QIBA Diffusion Profile tolerances. Volunteer‐based measurements showed higher variability in reproducibility, with sequence selection and motion as the most likely contributing factors. This study emphasizes the importance of completing qMRI QA both on phantoms and in vivo prior to commencing multi‐center clinical trials, including qMRI biomarkers to ensure data are accurate and reproducible. This study quantifies the variability in ADC measurement and is the first step to collecting reliable ADC datasets across Australian MRLs which will ideally lead to using these biomarkers in clinical applications such as disease characterization and treatment response monitoring.

## CONFLICT OF INTEREST STATEMENT

GenesisCare and Elekta AB (Stockholm, Sweden) have a strategic research agreement which includes financial support relating to the presented work.

## NIST DISCLAIMER

Certain commercial equipment, instruments, software or materials are identified in this paper in order to specify the experimental procedure adequately. Such identification is not intended to imply recommendation or endorsement by NIST, nor is it intended to imply that the materials or equipment identified are necessarily the best available for the purpose. NIST researchers were not involved in the human subjects research.

## Supporting information



Supporting information

Supporting information

Supporting information

Supporting information

Supporting information

## Data Availability

Authors will share data upon reasonable request to the corresponding author.

## References

[1] Padhani AR , Liu G , Mu‐Koh D , et al. Diffusion‐weighted magnetic resonance imaging as a cancer biomarker: consensus and recommendations. In: Neoplasia. Vol. 11. Elsevier B.V.; 2009:102‐125. doi:10.1593/neo.81328 PMC2631136 19186405

[2] El‐Habashy DM , Wahid KA , He R , et al. Longitudinal diffusion and volumetric kinetics of head and neck cancer magnetic resonance on a 1.5 T MR‐linear accelerator hybrid system: a prospective R‐IDEAL stage 2a imaging biomarker characterization/pre‐qualification study. Clin Transl Radiat Oncol. 2023;42:1‐10. doi:10.1016/j.ctro.2023.100666 PMC10424120 37583808

[3] Yang Y , Cao M , Sheng K , et al. Longitudinal diffusion MRI for treatment response assessment: preliminary experience using an MRI‐guided tri‐cobalt 60 radiotherapy system. Med Phys. 2016;43(3):1369‐1373. doi:10.1118/1.4942381 26936721 PMC6961701

[4] van Houdt PJ , Yang Y , Van Der Heide UA . Quantitative magnetic resonance imaging for biological image‐guided adaptive radiotherapy. Front Oncol. 2021;10:1‐9. doi:10.3389/fonc.2020.615643 PMC7878523 33585242

[5] Kooreman ES , van Houdt PJ , Nowee ME , et al. Feasibility and accuracy of quantitative imaging on a 1.5 T MR‐linear accelerator. Radiother Oncol. 2019;133:156‐162. doi:10.1016/j.radonc.2019.01.011 30935572

[6] Lawrence LSP , Chan RW , Chen H , et al. Accuracy and precision of apparent diffusion coefficient measurements on a 1.5 T MR‐Linac in central nervous system tumour patients. Radiother Oncol. 2021;164:155‐162. doi:10.1016/j.radonc.2021.09.020 34592363

[7] Shukla‐Dave A , Obuchowski NA , Chenevert TL , et al. Quantitative imaging biomarkers alliance (QIBA) recommendations for improved precision of DWI and DCE‐MRI derived biomarkers in multicenter oncology trials. J Magn Resonan Imaging. 2019;49(7):e101‐e121. doi:10.1002/jmri.26518 PMC6526078 30451345

[8] Quantitative Imaging Biomarkers Alliance . QIBA Profile: Diffusion‐Weighted Magnetic Resonance Imaging (DWI) ; 2019. https://qibawiki.rsna.org/images/7/7e/QIBADWIProfile_as_of_2019‐Feb‐05.pdf

[9] van Houdt PJ , Saeed H , Thorwarth D , et al. Integration of quantitative imaging biomarkers in clinical trials for MR‐guided radiotherapy: conceptual guidance for multicentre studies from the MR‐Linac Consortium Imaging Biomarker Working Group. Eur J Cancer. 2021;153:64‐71. doi:10.1016/j.ejca.2021.04.041 34144436 PMC8340311

[10] McDonald . In Vivo and Phantom Repeatability of Diffusion‐Weighted MRI Sequences on 1.5T MRI‐Linear Accelerator (MR‐Linac) and MR Simulator Devices for Head and Neck Cancers: Results from a Prospective R‐IDEAL Stage 2a Evaluation of Tumor and Normal Tissue Apparent Diffusion Coefficients as Quantitative Imaging Biomarkers; 2022.

[11] Habrich J , Boeke S , Nachbar M , et al. Repeatability of diffusion‐weighted magnetic resonance imaging in head and neck cancer at a 1.5 T MR‐Linac. Radiother Oncol. 2022;174:141‐148. doi:10.1016/j.radonc.2022.07.020 35902042

[12] Kooreman ES , van Houdt PJ , Keesman R , et al. ADC measurements on the Unity MR‐linac—a recommendation on behalf of the Elekta Unity MR‐linac consortium. Radiother Oncol. 2020;153:106‐113. doi:10.1016/j.radonc.2020.09.046 33017604 PMC8327388

[13] Subashi E , Dresner A , Tyagi N . Longitudinal assessment of quality assurance measurements in a 1.5 T MR‐linac: part II—Magnetic resonance imaging. J Appl Clin Med Phys. 2022;23(6):e13586. doi:10.1002/acm2.13586 PMC9398228 35332990

[14] Bisgaard ALH , Keesman R , van Lier ALHMW , et al. Recommendations for improved reproducibility of ADC derivation on behalf of the Elekta MRI‐linac consortium image analysis working group. Radiother Oncol. 2023;186:109803. doi:10.1016/j.radonc.2023.109803 PMC11197850 37437609

[15] CaliberMRI . Diffusion Phantom for ADC QMRI Standardization: Revision G; 2021. Accessed September 9, 2021. https://qmri.com/diffusion‐phantom‐manual‐spec‐sheet/

[16] Boss MA , Chenevert TL , Waterton JC , et al. Temperature‐Controlled Isotropic Diffusion Phantom with Wide Range of Apparent Diffusion Coefficients for Multicenter Assessment of Scanner Repeatability and Reproducibility; 2014. https://archive.ismrm.org/2014/4505.html

[17] Ingle M , Blackledge M , White I , et al. Quantitative analysis of diffusion weighted imaging in rectal cancer during radiotherapy using a magnetic resonance imaging integrated linear accelerator. Phys Imaging Radiat Oncol. 2022;23:32‐37. doi:10.1016/j.phro.2022.06.003 35756883 PMC9214864

[18] Kerkmeijer LGW , Fuller CD , Verkooijen HM , et al. The MRI‐linear accelerator consortium: evidence‐based clinical introduction of an innovation in radiation oncology connecting researchers, methodology, data collection, quality assurance, and technical development. Front Oncol. 2016;6:215. doi:10.3389/fonc.2016.00215 PMC5061756 27790408

[19] Carr ME , Keenan KE , Rai R , et al. Conformance of a 3T radiotherapy MRI scanner to the QIBA Diffusion Profile. Med Phys. 2022;49(7):4508‐4517. doi:10.1002/mp.15645 35365884 PMC9543906

[20] Quantitative Imaging Biomarkers Alliance . QIBA Profile Conformance Testing DWI MR Supplement 1. Accessed August 24, 2024. https://qibawiki.rsna.org/images/5/54/QIBA_DWI_Profile_Conformance_Testing_Supplement_1_v2023nov2_nonQIDW.pdf

[21] CaliberMRI . QCal MR: Automate the Known for Confident MRI Standardization; 2022. Accessed May 23, 2022. https://qmri.com/qmri‐platform/qcal‐software/

[22] Le Bihan D , Breton E , Lallemand D , Aubin ML , Vignaud J , Laval‐Jeantet M . Separation of diffusion and perfusion in intravoxel incoherent motion MR imaging. Radiology. 1988;168(2):497‐505. doi:10.1148/radiology.168.2.3393671 3393671

[23] Chenevert TL , Malyarenko DI , Newitt D , et al. Errors in quantitative image analysis due to platform‐dependent image scaling. Transl Oncol. 2014;7(1):65‐71. doi:10.1593/tlo.13811 24772209 PMC3998685

[24] Keenan KE , Jordanova KV , Ogier SE , et al. Phantoms for Quantitative Body MRI: a review and discussion of the phantom value. MAGMA. 2024;37(4):535–549. doi:10.1007/s10334-024-01181-8 38896407 PMC11417080

[25] QIBA DWI Biomarker Committee . QIBA Profile: Magnetic Resonance Diffusion‐Weighted Imaging (DWI) of the Apparent Diffusion Coefficient (ADC), Clinically Feasible Version; 2022. Accessed October 26, 2024. doi:10.1148/QIBA/20221215

[26] Newitt DC , Malyarenko D , Chenevert TL , et al. Multisite concordance of apparent diffusion coefficient measurements across the NCI Quantitative Imaging Network. J Med Imaging. 2017;5(01):1. doi:10.1117/1.jmi.5.1.011003 PMC5633866 29021993

[27] Tyagi N , Cloutier M , Zakian K , Deasy JO , Hunt M , Rimner A . Diffusion‐weighted MRI of the lung at 3T evaluated using echo‐planar‐based and single‐shot turbo spin‐echo‐based acquisition techniques for radiotherapy applications. J Appl Clin Med Phys. 2019;20(1):284‐292. doi:10.1002/acm2.12493 30421496 PMC6333125

