# Supplementary material for: Quantifying multi‐institutional ADC measurement variability of 1.5 T MR‐Linacs: A phantom and in vivo study
Source: Med Phys. 2025 Mar 13;52(6):4120–33. doi: 10.1002/mp.17739 (PMC12149690; doi:10.1002/mp.17739)
Supplement: Supplementary file 1 — Supporting information [file MP-52-4120-s005.pdf]

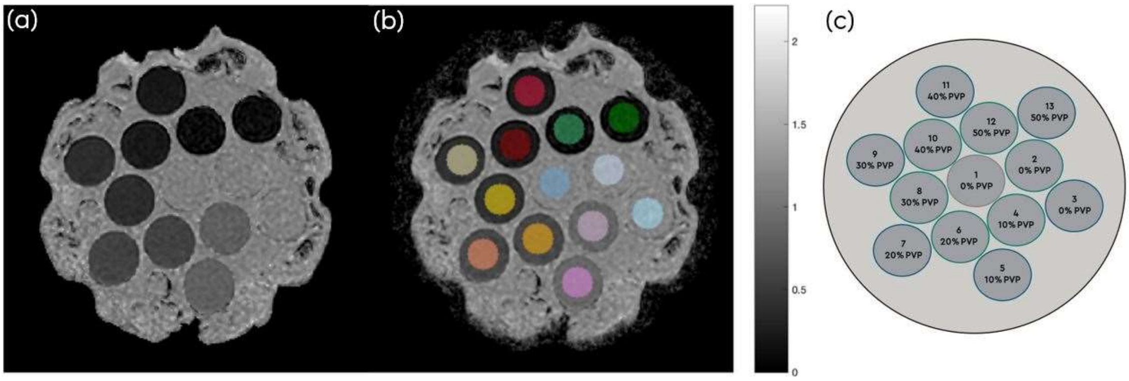

Supplementary Figure 1. (a) Inline and (b) offline ADC maps (with overlaid ROIs generated by qCal), both in units of $\mu m^2/ms$, of the phantom acquired using the QIBA EPI sequence for site A, day 1, repetition 1. Also, (c) shows a schematic of the diffusion phantom, highlighting the vial number and corresponding PVP concentrations. Note: The distortions occurring around vial 11 are visible, along with distortions in the outer regions of the phantom. This is especially the case at the anterior surface (top of the image), likely attributed to air-bubbles in the ice-bath which are likely to migrate to the top phantom surface when in its axial orientation in the scanner.
