# Supplementary material for: Quantifying multi‐institutional ADC measurement variability of 1.5 T MR‐Linacs: A phantom and in vivo study
Source: Med Phys. 2025 Mar 13;52(6):4120–33. doi: 10.1002/mp.17739 (PMC12149690; doi:10.1002/mp.17739)
Supplement: Supplementary file 2 — Supporting information [file MP-52-4120-s004.pdf]

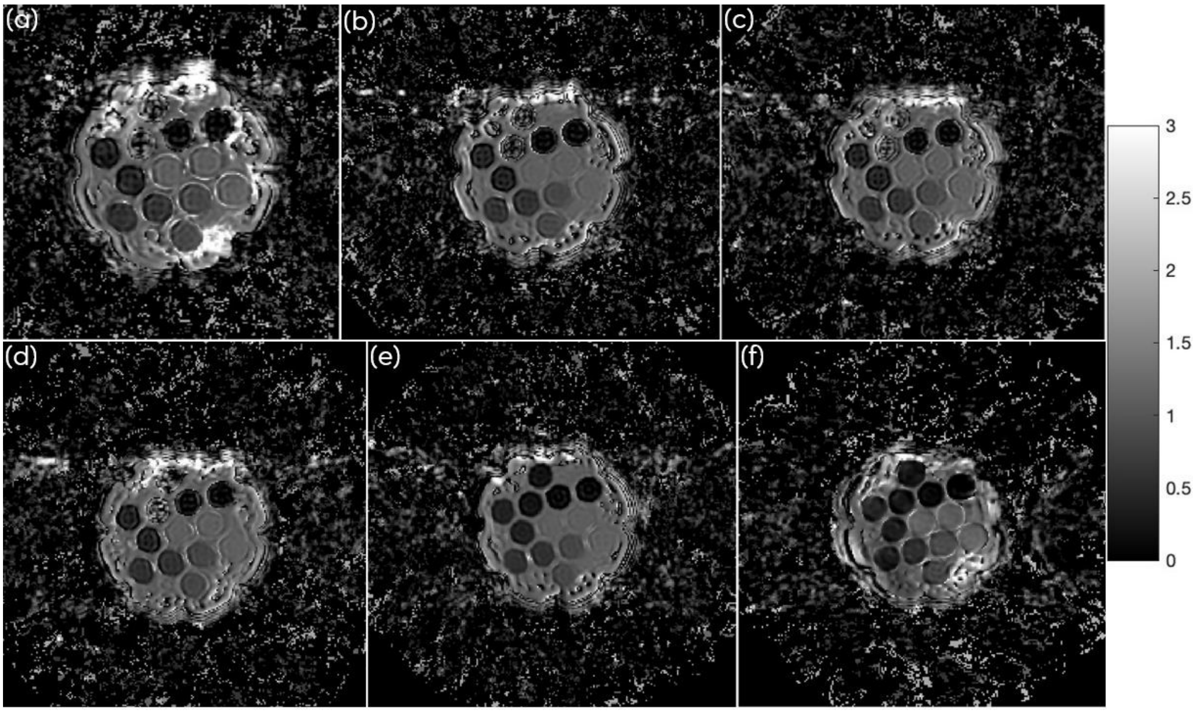

Supplementary Figure 2. Inline ADC maps (in units of µm²/ms) for each Consortium sequence for site A, day 1, including: (a) head and neck, (b) lung, (c) oesophagus, (d) pancreas/lymph, (e) prostate, and (f) cervix/rectum. Notice the distinct signal deficits in vials 10 and 11, causing significant deviations in ADC value per ROI in (a)-(d). Additionally, vial/phantom geometric distortions can be observed for all sequences with the phantom in its axial orientation in the scanner, especially for outer-ring vials and at the anterior (top) phantom surface. Note: Images (b) and (d) were cropped in the above image and do not show the full field of view. However, no images have been re-scaled relative to each other.
