# Supplementary material for: Quantifying multi‐institutional ADC measurement variability of 1.5 T MR‐Linacs: A phantom and in vivo study
Source: Med Phys. 2025 Mar 13;52(6):4120–33. doi: 10.1002/mp.17739 (PMC12149690; doi:10.1002/mp.17739)
Supplement: Supplementary file 3 — Supporting information [file MP-52-4120-s001.pdf]

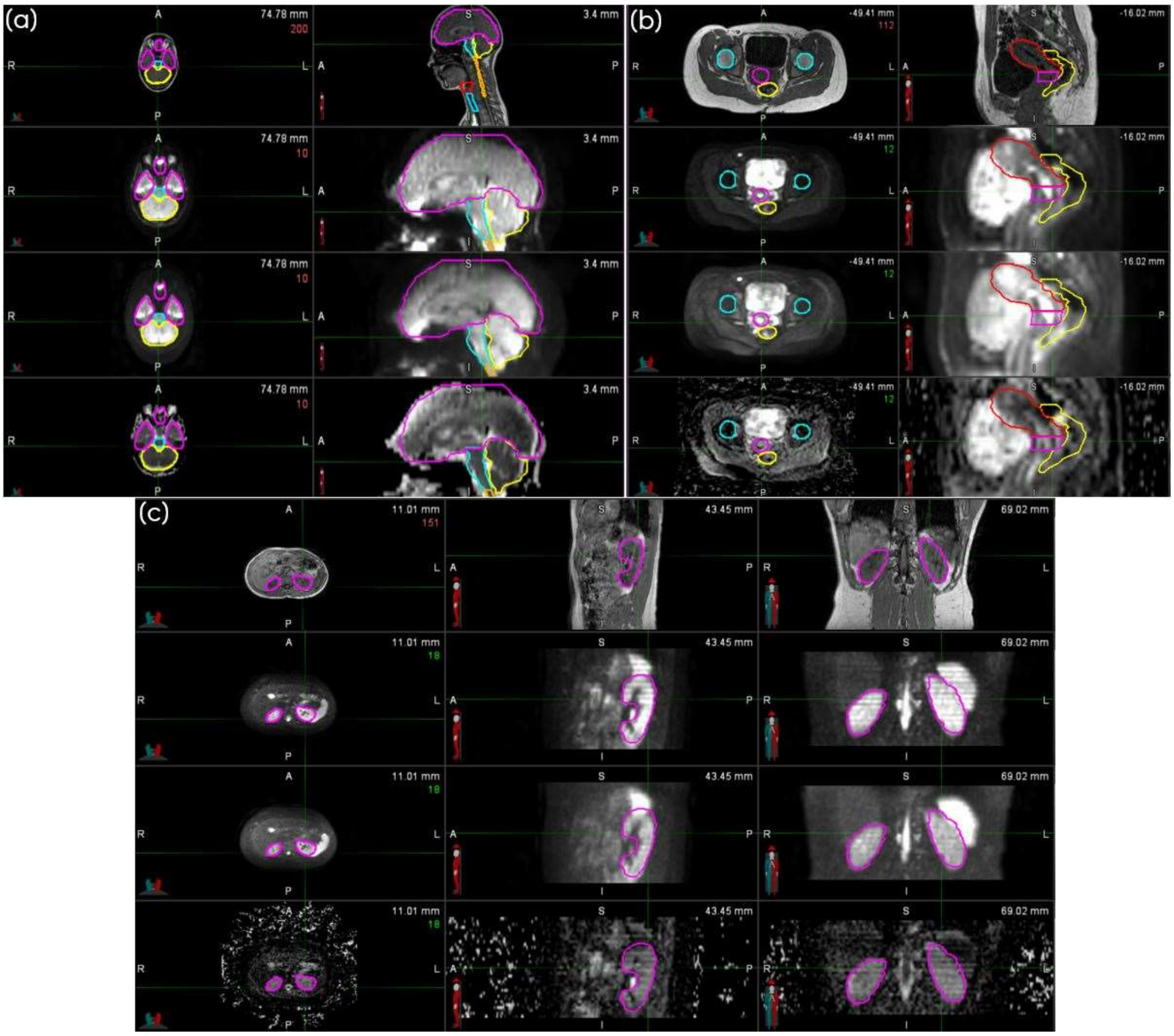

Supplementary Figure 3. Example volunteer images acquired from site A for the following anatomical regions (and sequences) (a) brain (brain DWI), (b) pelvis (Consortium cervix), and (c) abdomen (Consortium pancreas). In each case, top-to-bottom, the images were acquired using the following respective sequences: 3D T1-weighted, DW-images with b-values = 150 and 500 s/mm^2, and finally the corresponding inline ADC-maps. Contours from each anatomical region are included where visible, although not all contours were able to be used for analysis due to being outside the field of view of the ADC maps. For brain, the included contours in the analysis were brain (pink), cerebellum (yellow), and brainstem (blue). For the pelvis, this included contours of the femurs (blue), rectum (yellow), and uterus (red), cervix (pink). And lastly, for the abdomen, this included the kidneys (left and right combined – pink).
