# Supplementary material for: Quantifying multi‐institutional ADC measurement variability of 1.5 T MR‐Linacs: A phantom and in vivo study
Source: Med Phys. 2025 Mar 13;52(6):4120–33. doi: 10.1002/mp.17739 (PMC12149690; doi:10.1002/mp.17739)
Supplement: Supplementary file 4 — Supporting information [file MP-52-4120-s003.pdf]

Supplementary Table 1. Overview of QIBA diffusion Profile tests and calculations performed in this study to assess intra- and inter-scanner Profile conformance. Note that excluding test D, Profile testing was only required to be performed on the central water vial (at isocenter). Further, short-term (ST), refers to the intra-day intra-scanner measurements, whilst between-session (BS) refers to the intra-scanner measurements acquired using the first QIBA sequence measurements on two consecutive days from 1 MRL at a time. Profile tolerance values can be found in Table 2. Table adapted from Carr et al. (2022).

Test	Relevant Equations	Definitions	
**A**	$\lvert \%bias \rvert = \left(\frac{\mu - DC_T}{DC_T}\right) \times 100\%$	DC_T<br>μ	= True diffusion coefficient<br>= Mean of measurements
**B/C**	$RC_{ST/BS} = 2.77 \times SD$<br><br>$CV_{ST/BS} = 100\% \times \frac{SD}{\mu}$	RC<br>$CV_{ST/BS}$<br><br>SD	= Repeatability coefficient<br>= Intra-scanner coefficient of variation in %bias over 4 x short-term (ST), OR 2 x between-session (BS) measurements<br>= Standard deviation
**D**	$R^2 = 1 - \frac{RSS}{TSS}$<br>AND<br>$Y = \beta_0 + \beta_1 \times DC_T$	R^2<br>RSS<br>TSS<br>Y<br>β_0<br>β_1	= Coefficient of determination<br>= Sum of squares of residuals<br>= Total Sum of Squares<br>= Measured ADC (all vials/months)<br>= Intercept<br>= Slope
**E**	$CV_P = 100\% \times \frac{SD_{pix}}{\mu_{ROI}}$	ROI<br>SD_{pix}	= Region of interest (isocenter vial)<br>= Over ADC values within the ROI
**F**	$SNR = \frac{\mu_{ROI}\,[Signal\ Image]}{\mu_{ROI}\,[Noise\ Image]}$	SNR<br>Signal<br>Noise	= Signal to noise ratio<br>= Average of pixel values for each ROI over the 4 x ST repetitions<br>= Average of pixel SD values for each ROI over the 4 x ST repetitions
**G**	$Dep_b = 100\% \times \left\lvert \frac{ADC_{b0,bn+1} - ADC_{b0,bn}}{ADC_{b0,bn}} \right\rvert$	Dep_b<br>$ADC_{b0,bn+1}$<br><br>b_{1-3}	= *b*-value dependence<br>= ADC generated using b_0 = 0 s/mm^2 and b_{n+1}, where $b_{n+1} > b_n$<br>= 500, 900 or 2000 s/mm^2
**H**	$CV = 100\% \times \frac{SD_{inter-scanner}}{\mu_{\ inter-scann}}$	CV	= Coefficient of variation for inter-scanner reproducibility. For the QIBA Diffusion sequences, qCal utilized the average %bias from the 4x repetitions on day 1 on each respective MRL to then calculate the inter-scanner μ and SD. This differs to the Python based analysis on the Consortium sequences, which utilized only the singular day 1 ADC values from each scanner/sequence.
