# Supplementary material for: Quantifying multi‐institutional ADC measurement variability of 1.5 T MR‐Linacs: A phantom and in vivo study
Source: Med Phys. 2025 Mar 13;52(6):4120–33. doi: 10.1002/mp.17739 (PMC12149690; doi:10.1002/mp.17739)
Supplement: Supplementary file 5 — Supporting information [file MP-52-4120-s002.pdf]

Supplementary Table 2. Organs with ADC values able to be successfully extracted from each dataset acquired between the three MRL sites (✓). Other contoured organs, such as the larynx, parotids, spinal cord, and trachea, were excluded from analysis. These organs were not fully contained within the ADC map contours (based on the T1-weighted images), for one or more occurrences at all the three sites, making them unsuitable for reproducibility assessments.

	**Site A**		**Site B**		**Site C**	
**Volunteer organs included in analysis**	**Day 1**	**Day 2**	**Day 1**	**Day 2**	**Day 1**	**Day 2**
**Brain**	✓	✓	✓	N/A	✓	N/A
**Brainstem**	✓	✓	✓	N/A	✓	N/A
**Cerebellum**	✓	✓	✓	N/A	✓	N/A
**Orbits**	✓	✓	✓	N/A	✓	N/A
**Kidneys**	✓	✓	✓	✓	✓	N/A
**Cervix**	✓	✓	✓	✓	✓	✓
**Femurs**	✓	✓	✓	✓	✓	✓
**Rectum**	✓	✓	✓	✓	✓	✓
**Uterus**	✓	✓	✓	✓	✓	✓
